# Supplementary material for: In Silico drug repurposing pipeline using deep learning and structure based approaches in epilepsy
Source: Sci Rep. 2024 Jul 17;14:16562. doi: 10.1038/s41598-024-67594-6 (PMC11254927; doi:10.1038/s41598-024-67594-6)
Supplement: Supplementary file 1 — Supplementary Information 1. [file 41598_2024_67594_MOESM1_ESM.pdf]

In Silico Drug Repurposing Pipeline using Deep Learning and Structure based Approaches in Epilepsy

Xiaoying Lv[1], Jia Wang[2], Ying Yuan[1], Lurong Pan[1], Qi Liu[1] Jinjiang Guo[1*]

[1] Global Health Drug Discovery Institute, Beijing, China

[2] Cipher Gene Limited, Beijing, China

* corresponding author

Email: jinjiang.guo@ghddi.org

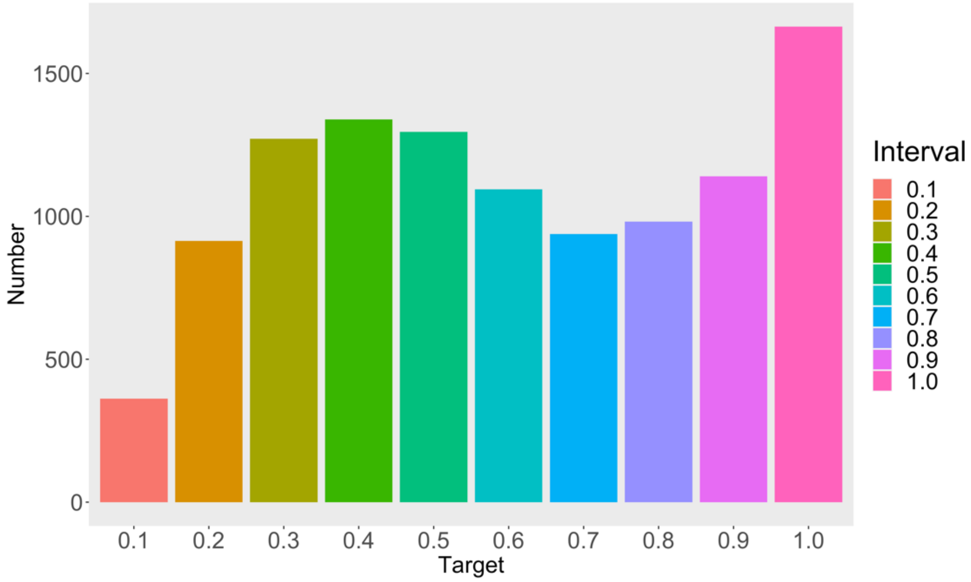

Fig S1 The distribution of predicted BBB permeability probability for compounds from DrugBank database.

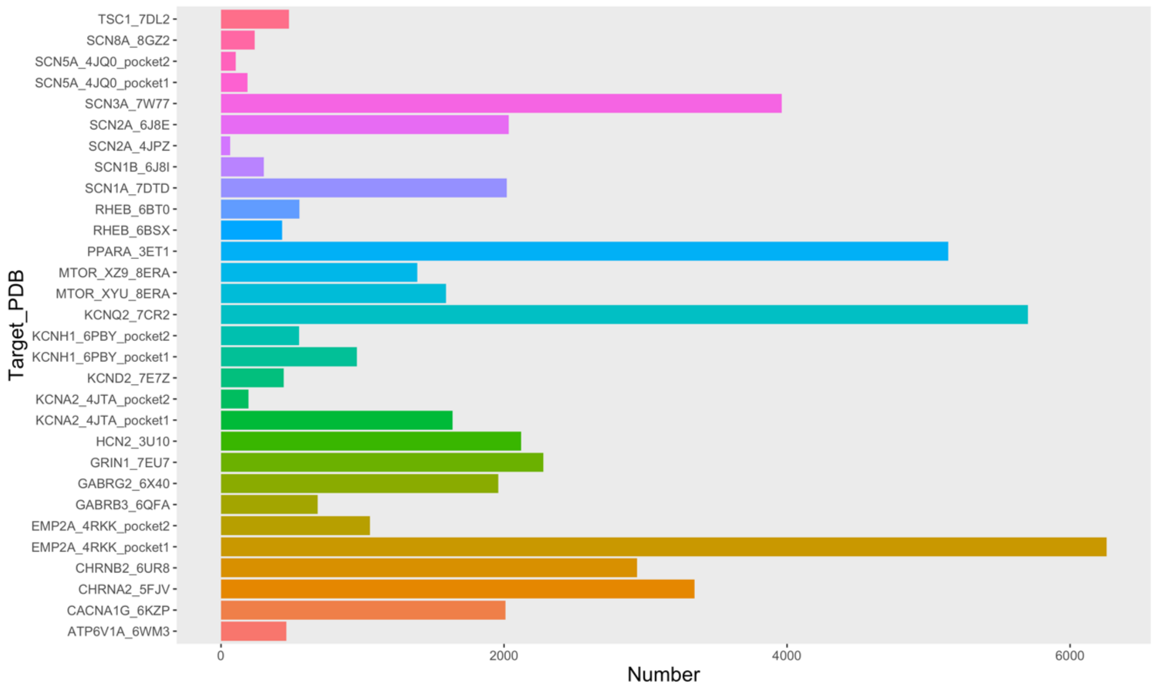

Fig S2 The number of candidates after docking filter using -7.0 as threshold criterion.

a b

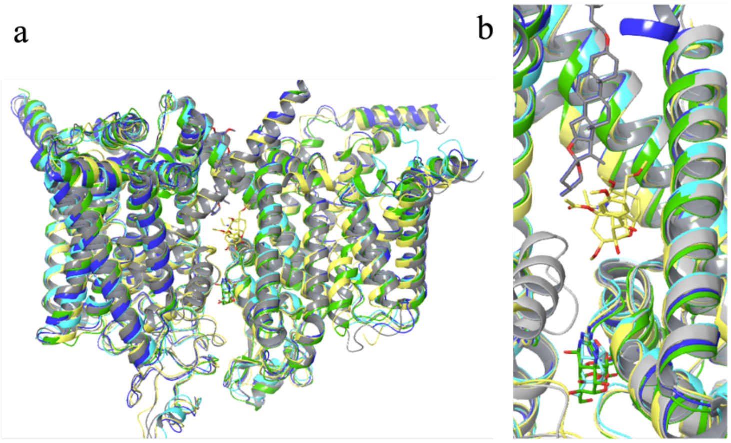

Fig S3 The five voltage-gated sodium channel structures. a. Using SCN1A (7DTD:gray) as a reference structure, the other four structures were aligned using protein (cartoon). The RMSD for SCN2A (6J8E:blue), SCN3A (7W77:yellow), SCN8A (8GZ2:cyan) and SCN9A (6J8I:green) are 1.664 Å, 1.373 Å, 1.779 Å and 1.664 Å, respectively. b. The close-up view of panel a. These ligands are represented by stick with the same color of corresponding protein in panel a.

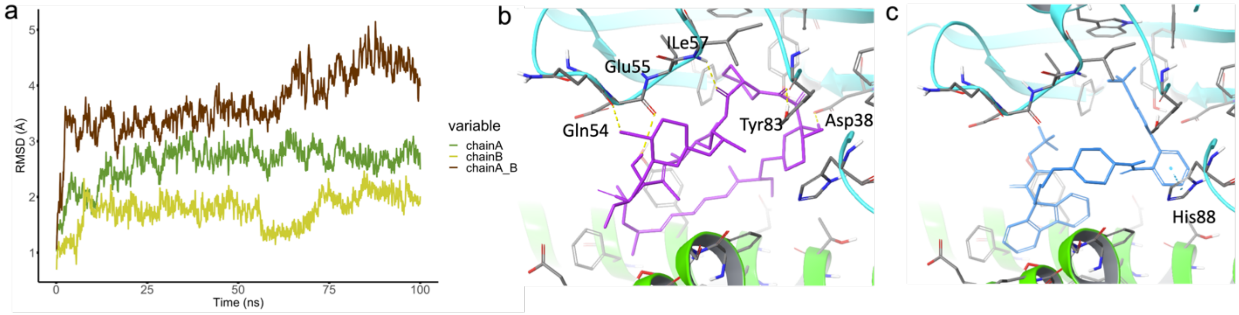

Fig S4 The RMSD plot and ligand-receptor interaction for the target (PDB ID: 8ERA). Panel a, RMSD plot for the protein backbone, calculated across 8ERA complex and its chain A and B during the 100ns production phase. In panel b and c, chain A encoded by *MTOR* gene and chain B encoded by *FKBP1A* gene was highlighted in green and cyan, respectively. The co-crystal XYU (panel b) and Lomitapide (panel c) were colored by purple and blue, respectively.

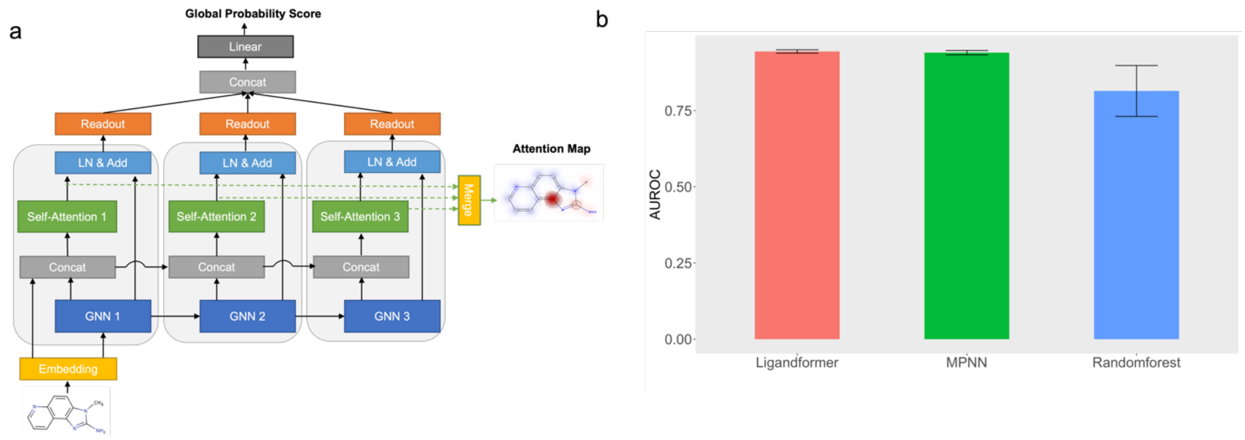

Fig S5 Ligandformer architecture and performance comparison with MPNN and Random forest model. a) This is a multi-layer single-head self-attention-based Graph Neural Network framework, for predicting compound chemical property with robust interpretation. Facilitated with visualization technique, the map shows insights on AI model's rationales on judging which parts of an input molecule impact certain property predictions. b) The AUROC performance of Ligandformer, MPNN and Randomforest model in B3DB database are colored by red, green and blue, respectivley. The value of vertical axis is Aera Under ROC and error bar was shown by black line.

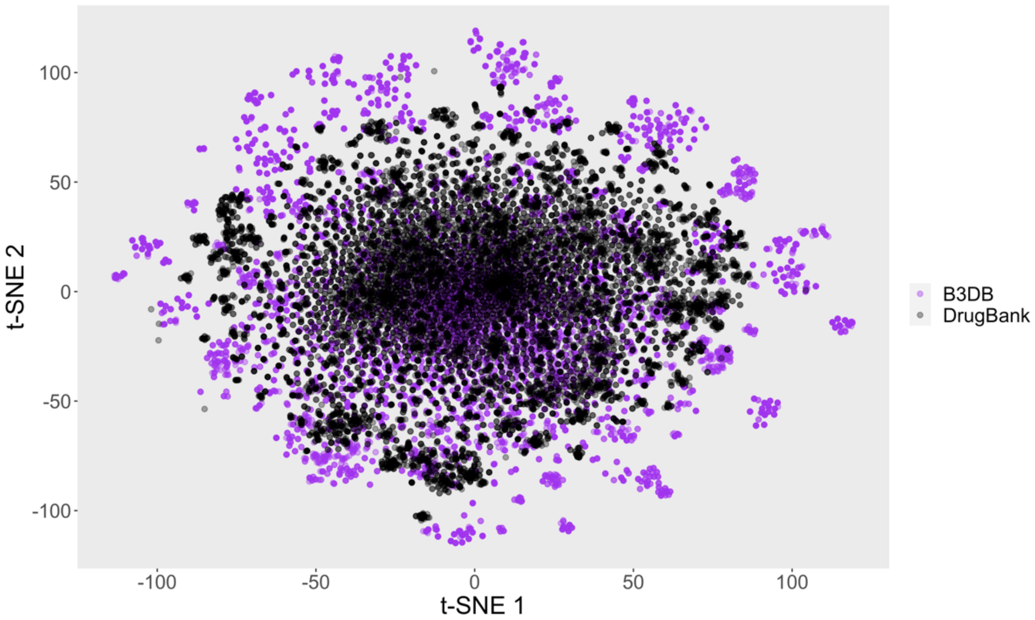

Fig S6 The t-SNE chemical space of compounds from B3DB (purple dot) and Drugbank (black dot) databases.

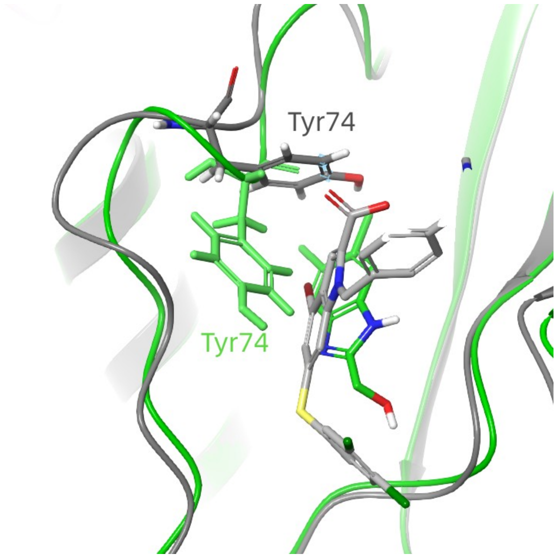

Fig S7 The two conformations of Tyr74 in 6BT0 (gray) and 6BSX (green) crystal structures. For RHEB target, the binding poses of ligand E7V (gray) and E7S (green) are shown.

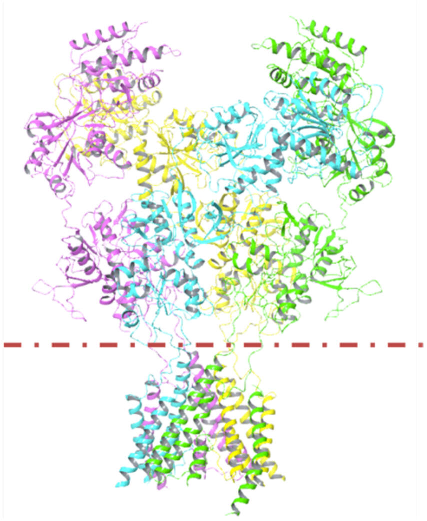

Fig S8 The structure of 7EU7. Only transmembrane region was retained in 100-ns MD simulation (below dotted line.)

Table S1 The BBB permeable probability of anti-epileptic drugs in market predicted by Ligandformer model

**Drug**	**ID**	**Probability**
Carbamazepine	DB00564	0.9117
Valproic acid	DB00313	0.9282
Phenytoin sodium	DB00252	0.8325
Oxcarbazepine	DB00776	0.94
Diazepam	DB00829	0.97
Levetiracetam	DB01202	0.95
Fosphenytoin	DB01320	0.8445
Flunarizine	DB04841	0.9560

Movie S1-S10 The 100-ns MD simulation for lomitapide in EMP2A (PDB ID: 4RKK), CHRNA2 (PDB ID: 5FJV), CACNA1G (PDB ID: 6KZP), KCNH1 (PDB ID: 6PBY), GABRB3 (PDB ID: 6QFA), SCN1A (PDB ID: 7DTD), KCND2 (PDB ID: 7E7Z), GRIN1 (PDB ID: 7EU7), SCN3A (PDB ID: 7W77), MTOR (PDB ID: 8ERA) targets.
